# Implication of orexin transmission in chronic stress, hyperarousal, and neuroplasticity deficits: a mechanistic framework for depression and anxiety

**DOI:** 10.3389/fpsyt.2026.1960782

**Published:** 2026-09-28

**Authors:** Alexa C. E. Alcala, Sophia C. Bellitire, Katherine E. Brunson, Sophia dV. Kuper, Rémi Martin-Fardon, Francisco J. Flores-Ramirez

**Affiliations:** 1 Department of Psychology, California State University San Marcos, San Marcos, CA, United States; 2 Department of Translational Medicine, The Scripps Research Institute, San Diego, CA, United States

**Keywords:** anxiety, corticotropin- releasing factor (CRF), hyperarousal, orexin (hypocretin), stress

## Abstract

Stress is a major risk factor for depression- and anxiety-related disorders and produces neuroplastic adaptations within brain regions that regulate emotion, cognition, and stress responsivity. These adaptations include dendritic retraction in the medial prefrontal cortex (mPFC), suppression of plasticity and neurogenesis in the hippocampus (HIPP), and hypertrophic remodeling of circuits within the amygdala (AMY), promoting emotional dysregulation and maladaptive behavior. Although these alterations are well-documented, the mechanisms that coordinate their emergence across multiple neural systems remain unknown. The orexin (OX; hypocretin) system is anatomically and functionally positioned to serve as a key mediator of stress-induced neuroplasticity. Indeed, OX neurons receive direct corticotropin-releasing factor (CRF) input from the hypothalamic paraventricular nucleus (PVN) and project broadly to brain regions involved in arousal, motivation, emotional regulation, and reward processing (e.g., mPFC, HIPP, and AMY). Chronic activation of CRF-sensitive OX pathways may therefore sustain OX-dependent hyperarousal beyond the presence of an acute stressor, increasing excitatory drive throughout emotion-regulation circuits. This prolonged activation may contribute to maladaptive plasticity through alterations in neuronal excitability, impaired homeostatic regulation, and reductions in brain-derived neurotrophic factor (BDNF)-related signaling. In this review, we propose a mechanistic framework in which CRF-mediated recruitment of hypothalamic OX neurons contributes to neuroplasticity deficits in the mPFC, HIPP, and AMY. We discuss how developmental stage, biological sex, and genetic variation influence vulnerability to these adaptations and examine therapeutic strategies aimed at reducing hyperarousal and restoring neural plasticity. Collectively, this theoretical framework positions OX transmission as a potential link between chronic stress and mood-related psychopathology.

## 1 Overview

In this review, we propose a theoretical mechanistic framework in which chronic stress-induced activation of corticotropin-releasing factor (CRF) systems repeatedly recruits orexin (OX; hypocretin) neurons within the lateral hypothalamus (LH), dorsomedial hypothalamus (DMH), and perifornical area (PFA). We hypothesize that sustained CRF-mediated excitation of these neurons increases OX transmission throughout stress-sensitive corticolimbic circuits, including the medial prefrontal cortex (mPFC), hippocampus (HIPP), amygdala (AMY), and associated reward networks, where OX receptor signaling may contribute to persistent arousal states and maladaptive neuroplastic remodeling. We further discuss developmental, biological, and genetic moderators of vulnerability and examine emerging therapeutic strategies aimed at restoring neural plasticity and reducing pathological hyperarousal. By integrating evidence across molecular, cellular, circuit, and behavioral levels, we argue that CRF→OX transmission may provide a useful framework for understanding how chronic stress is translated into depression- and anxiety-related psychopathology. This conceptual pathway is summarized in **Figure 1**, which depicts the proposed interactions among CRF signaling, OX activation-induced hyperarousal, and downstream neuroplasticity changes.

**Figure 1:**
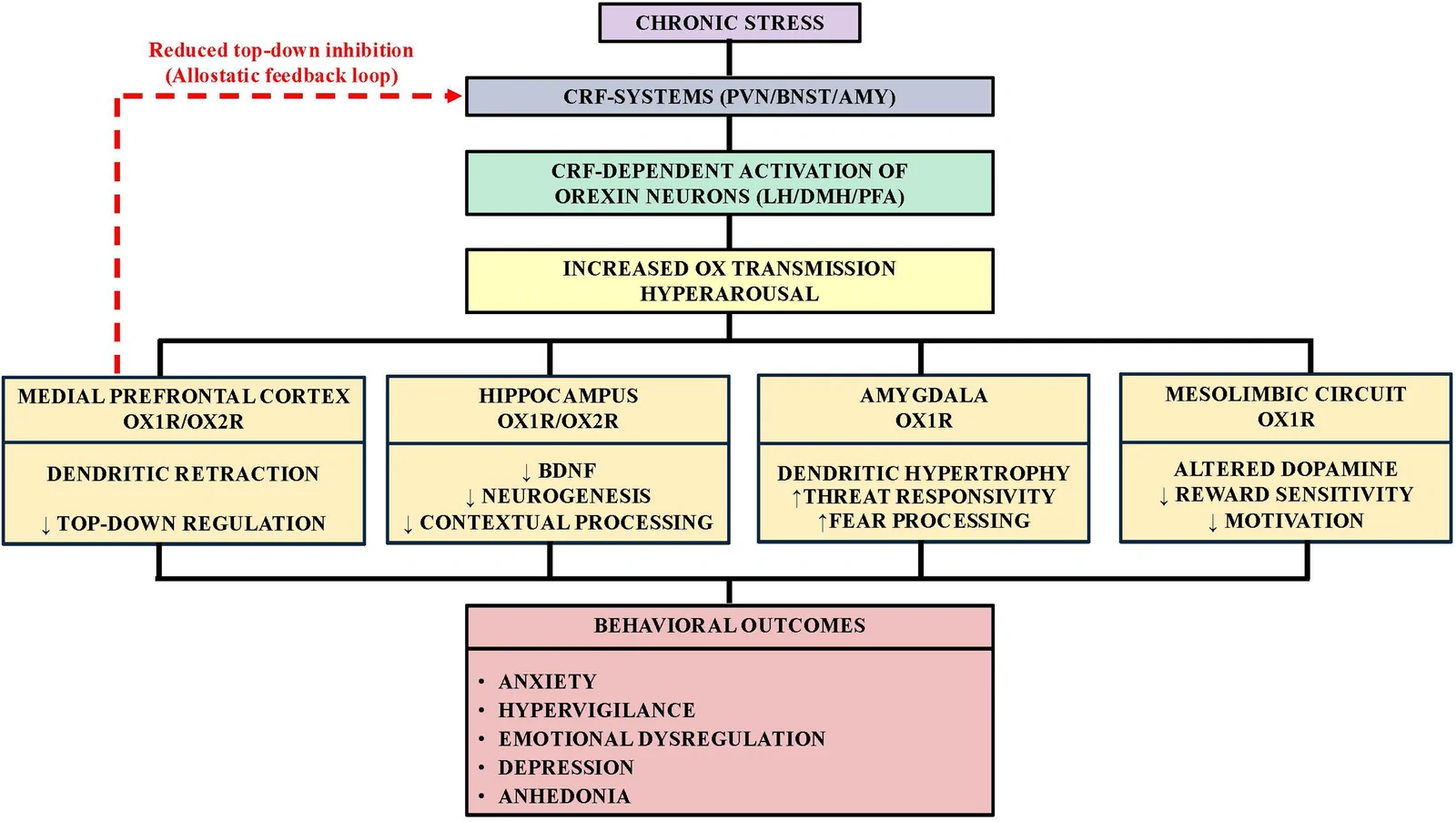
Proposed model linking chronic stress, corticotropin-releasing factor (CRF)-mediated recruitment of orexin (OX) neurons, hyperarousal, and neuroplasticity deficits associated with depression- and anxiety-related psychopathology. Chronic stress recruits CRF signaling within the paraventricular nucleus (PVN), bed nucleus of the stria terminalis (BNST), and amygdala (AMY). These stress-responsive CRF systems activate OX neurons within the lateral hypothalamus (LH), dorsomedial hypothalamus (DMH) and perifornical area (PFA), resulting in increased OX transmission throughout stress-sensitive corticolimbic networks. Sustained CRF-mediated recruitment of OX neurons may promote persistent OX-dependent hyperarousal characterized by heightened vigilance, increased autonomic activation, and impaired recovery following stress exposure. Because OX neurons project broadly throughout the central nervous system, prolonged OX transmission may contribute to widespread circuit-level adaptations. Within the medial prefrontal cortex (mPFC) and hippocampus (HIPP), chronic stress is associated with dendritic retraction, reduced brain-derived neurotrophic factor (BDNF) signaling, impaired neurogenesis, and diminished neuroplasticity. In contrast, the amygdala (AMY) exhibits increased dendritic complexity and enhanced threat responsivity. Simultaneously, dysregulation of mesolimbic reward circuitry may contribute to altered dopaminergic signaling and reduced reward sensitivity. In the proposed model, OX transmission functions as an allostatic amplifier that extends the influence of chronic stress across multiple corticolimbic networks, potentially contributing to hypervigilance, emotional dysregulation, anxiety, depression, and anhedonia. In addition, impaired top-down regulation from the mPFC may further promote activation of stress-responsive CRF systems and hypothalamic OX neurons, creating a self-reinforcing allostatic state that sustains hyperarousal and maladaptive neuroplastic remodeling.

## 2 Background

Chronic stress is a major risk factor in the ontogeny of depression- and anxiety-related disorders and is associated with persistent alterations in brain structure and function. While acute stress responses are generally adaptive and promote survival, prolonged activation of stress-responsive systems can produce maladaptive neurobiological changes that extend beyond the termination of the stressor itself (1–3). One influential framework for understanding these effects is the concept of allostasis, which proposes that organisms maintain stability through adaptive physiological change (2, 4). While activation of stress-responsive systems can be beneficial in the short term, chronic recruitment of these systems promotes the accumulation of an “allostatic load” which can ultimately contribute to pathology (3). In the central nervous system, this burden is associated with widespread neuroplastic adaptations affecting stress-sensitive corticolimbic structures, including the mPFC, HIPP, and AMY. These adaptations are thought to contribute to the cognitive, emotional, and behavioral abnormalities that are characteristic of stress-related psychiatric disorders (3, 5).

Many of these adaptations reflect disruptions in neural plasticity, the capacity of neural circuits to modify their structure and function in response to experience (6). Beyond synaptic modification, chronic stress produces profound structural remodeling throughout corticolimbic circuitry, wherein repeated exposure to stress results in dendritic retraction and reduced synaptic complexity within the mPFC and HIPP, while simultaneously promoting dendritic growth and enhanced excitability within the AMY (7–9). These opposing patterns of remodeling are accompanied by reductions in brain-derived neurotrophic factor (BDNF), a key regulator of neuronal survival, synaptic plasticity, and dendritic maintenance in the HIPP and mPFC (10, 11).

Although the structural, functional, and molecular consequences of chronic stress have been extensively characterized, the mechanisms that coordinate these adaptations across multiple brain regions are yet to be fully clarified. In addition to glucocorticoids, inflammatory markers, glutamatergic and monoaminergic mechanisms, growing evidence suggests that the OX system may occupy a unique position at the intersection of stress responsivity, arousal regulation, motivation, and neural plasticity (12–14). OX neurons receive direct CRF input from stress-responsive hypothalamic regions, including the paraventricular nucleus (PVN) and the bed nucleus of the stria terminalis (BNST), and project broadly throughout stress-sensitive corticolimbic circuitry, including the mPFC, HIPP, AMY, and mesolimbic reward system (15–18). Because OX neurons occupy a position downstream of CRF-mediated stress signaling while simultaneously influencing multiple corticolimbic targets, repeated stress-induced recruitment of CRF systems may provide a mechanism through which chronic stress is translated into persistent hyperarousal and widespread neuroplastic remodeling.

## 3 Chronic stress-induced OX activation: hyperarousal

The ability to rapidly detect and respond to environmental challenges is essential for survival (19). During acute stress, activation of the CRF system initiates a coordinated physiological response that promotes vigilance, attention, autonomic activation, and behavioral adaptation (1). Under normal conditions, these responses are transient and subside once the stressor is removed, allowing neural and endocrine systems to return to baseline. However, chronic or repeated stress exposure can disrupt this recovery process, producing a persistent state of hyperarousal characterized by sustained vigilance, sleep disturbances, heightened emotional reactivity, and impaired stress recovery (2–4).

Among the neural systems recruited during stress, the OX system is uniquely positioned to coordinate arousal-related responses across multiple brain regions. OX-producing neurons are localized primarily within the LH, DMH and perifornical area (PFA), yet send extensive projections throughout the central nervous system, including the mPFC, HIPP, AMY, locus coeruleus (LC), dorsal raphe nucleus, ventral tegmental area (VTA), and paraventricular nucleus of the hypothalamus (12, 17, 20). Through these widespread projections, OX has been proposed to influence arousal, stress, motivation, reward processing, autonomic regulation, attention, and emotional behavior, functions that support adaptive responses to environmental demands (17, 20, 21). Using electrophysiological recordings and immunohistochemical approaches in mice, it was demonstrated that CRF-containing terminals establish functional synaptic contacts with OX neurons in the LH and that CRF application increases OX neuronal firing (18). These findings identified a direct anatomical and functional pathway through which hypothalamic and extrahypothalamic CRF systems may recruit OX-dependent arousal networks. This interaction places OX neurons as key downstream effectors of stress-responsive CRF signaling, while simultaneously positioning them to influence a broad array of corticolimbic regions involved in emotional regulation and behavioral adaptation (12, 18).

Chronic stress is associated with sustained recruitment of both hypothalamic and extrahypothalamic CRF systems that extend beyond the acute termination of a stressor. In addition to activation of the PVN, repeated stress repeatedly engages CRF-containing neurons within the BNST and AMY, regions critically involved in anxiety-like behavior, autonomic regulation, and persistent stress responsivity (3, 12, 22, 23). Converging anatomical and electrophysiological evidence demonstrates that CRF-containing terminals establish functional synaptic contacts onto OX neurons and that CRF directly increases the firing rate of these cells (18). Consequently, repeated activation of CRF systems during chronic stress may result in sustained recruitment of OX neurons within the LH, DMH, and PFA (12, 24, 25). Because OX neurons project extensively to regions that regulate vigilance, autonomic output, emotional processing and behavioral responsiveness, prolonged activation of OX transmission has the potential to influence multiple components of the stress response simultaneously (12, 17, 20). Indeed, within the LC, OX promotes arousal and vigilance, whereas OX transmission within the AMY enhances threat-related processing and emotional reactivity (12, 26, 27). In corticolimbic regions such as the mPFC and HIPP, OX may further influence neuronal excitability, cognitive regulation, contextual processing, and stress related adaptation (12, 28). Collectively, these observations suggest that OX-dependent recruitment has the capacity to simultaneously engage neural systems governing vigilance, autonomic activation, threat detection and behavioral regulation. Because these regions operate as an integrated network, sustained OX transmission may promote a distributed state of hyperarousal characterized by heightened vigilance, increased autonomic activation, impaired recovery following stress exposure, and enhanced sensitivity to emotionally salient stimuli.

Under acute conditions, recruitment of OX signaling is likely adaptive. Indeed, increased OX release promotes wakefulness, attentional engagement, cardiovascular activation, and motivated behavior, facilitating an organism’s ability to respond effectively to environmental threats (21, 29, 30). However, repeated activation of this system during chronic stress may transform an adaptive response into a maladaptive state of sustained hyperarousal. Evidence supporting this notion comes from both clinical and preclinical studies demonstrating increased OX system activity during anxiety- and panic-related states. In a rat model of panic vulnerability using small-interfering RNA (siRNA) to suppress hypocretin (*Hcrt*) gene expression, animals were exposed to sodium lactate, a well-established panicogenic challenge. The results demonstrated *Hcrt* knockdown attenuated behavioral manifestations of panic-like responding, including reductions in social interaction and increased locomotor activity, while simultaneously preventing the associated cardiovascular responses, including tachycardia and elevated blood pressure (13). These findings suggest that OX transmission contributes to panic-related behavioral and autonomic dysregulation rather than simply reflecting a downstream consequence of stress exposure. Further, using optogenetic approaches in mice, it was shown that activation of OX neurons increases behavioral avoidance in anxiogenic environments, whereas disruption of OX signaling reduced anxiety-like responding in context-dependent manner (31). These observations suggest that OX may enhance the salience of environmental threats and promote behavioral states characterized by heightened vigilance. Consistent with this interpretation, chronic recruitment of the OX transmission contributes to pathological hyperarousal by increasing activity within neural systems that regulate attention, autonomic output, and emotional responsiveness (12, 13).

The transition from adaptive arousal to pathological hyperarousal may be further amplified by intrinsic properties of the OX network itself. Using whole-cell recordings from rat hypothalamic slices, application of Orexin-A (OX-A) and Orexin-B (OX-B), the two endogenous peptides, increased the activity of neighboring glutamatergic neurons, which subsequently excited OX-producing neurons and increased their firing rate by more than threefold relative to baseline (32). This local positive-feedback mechanism allows OX neurons to amplify their own activity and provides a cellular substrate through which repeated stress exposure may prolong OX transmission beyond the initial stressor. Thus, in the presence of persistent CRF-mediated excitation induced by chronic stress, for example, such feed-forward interactions could sustain elevated OX activity despite the absence of an initial environmental challenge. OX also exerts an influence over downstream circuits involved in arousal and emotional regulation. OX fibers densely innervate the locus coeruleus, where the activation of orexin receptors 1 (OX1Rs) increases noradrenergic neuronal firing and promotes vigilance and autonomic activation (12). In rats, OX-A depolarizes neurons within the AMY via OX1R-dependent mechanisms and pharmacological OX1R blockade reduces conditioned fear expression (26). These findings indicate that OX may modulate amygdalar circuitry involved in fear processing and emotional behavior.

Beyond its role in arousal and fear, OX also influences reward-related circuitry (33). OX neurons project to the VTA, where OX increases the activity of dopaminergic neurons and facilitates motivated behavior and reward seeking (29). Consequently, chronic hyperactivation of OX transmission has the potential to influence not only anxiety-related processes but also motivational and affective domains that are frequently disrupted in depression (34). In this context, recent conceptual evidence suggests that OX may contribute not only to acute stress responsivity, but also to the long-term remodeling of stress-responsive circuits. It has been proposed that OX may influence stress-related neuroplasticity through alterations in downstream gene expression, microcircuit organization, and circuit-level adaptation (14). This perspective extends the traditional view of OX as a wake-promoting neuropeptide (21) and suggest that maladaptive activation may contribute to structural and functional adaptations observed following prolonged stress exposure.

Although acute recruitment of OX neurons during stress is well-established (13, 18, 25, 35), a critical unresolved question is whether repeated stress exposure produces persistent alterations in OX system function that outlast the stressor itself. Several lines of evidence suggest that this may be the case. Exposure to a variety of stressors, including restraint stress, footshock, panicogenic stimuli, and predator-associated threats, increases OX neuronal activity and expression of immediate early genes within hypothalamic OX populations (13, 24, 25, 36). Likewise, activation of CRF-responsive circuits has been shown to recruit OX neurons directly, providing a mechanism through which repeated stress exposure may chronically engage OX-dependent arousal networks (18). Importantly, emerging evidence suggests that OX neurons themselves exhibit functional plasticity following repeated stress exposure, including alterations in excitability, peptide expression, and responsivity to subsequent stressors (14). Although direct evidence linking chronic stress-induced OX activation to the structural neuroplastic adaptations observed in the mPFC, HIPP, and AMY remains limited, these observations raise the possibility that sustained recruitment of OX neurons represents an intermediate mechanism through which repeated stress exposure influences long-term circuit remodeling. Consequently, OX transmission may function not only as a mediator of acute arousal, but also as an allostatic amplifier capable of extending the influence of chronic stress across distributed neural networks.

Collectively, the available evidence supports a model in which stress recruits CRF-sensitive OX neurons (24, 25), while local glutamatergic feedback mechanisms sustain and amplify OX network activity. Over time, repeated recruitment of CRF inputs onto OX neurons may promote lasting adaptations within stress-responsive systems, shifting neural function from adaptive stress regulation toward persistent states of hypervigilance, emotional dysregulation, and increased vulnerability to mood-related psychopathology (37). Because OX projections reach multiple corticolimbic regions simultaneously, this system is uniquely positioned to coordinate the widespread neuroplastic adaptations observed following stress.

## 4 Neuroplasticity as a downstream consequence of chronic stress

Neural plasticity refers to the ability of the nervous system to change its function and structure in response to environmental challenges and experience (38). These adaptations occur across multiple levels of biological organization, including changes in synaptic strength, dendritic architecture, neuronal excitability, gene expression, and adult neurogenesis, processes that allow neural circuits to encode experience, support learning and memory, and adapt to changing environmental conditions (39–41). While under normal conditions, plasticity promotes resilience by enabling organisms to respond adaptively to stress, chronic activation of stress responsive systems (e.g., CRF-signaling, inflammatory pathways, glutamatergic conditions, and OX-dependent arousal networks) can disrupt these processes and shift plasticity from an adaptive to a maladaptive state (3). A key observation is that one of the primary mechanisms through which chronic stress influences neural plasticity is sustained elevation of neuronal activity within stress-responsive circuits (42, 43). Recent evidence further demonstrates that chronic stress can disrupt the balance between excitatory and inhibitory signaling within stress-responsive neural circuits. For example, chronic stress-induced alterations in medial preoptic area (MPOA) circuitry were shown to produce persistent excitation-inhibition imbalance that contributed to depression-like behavioral phenotypes, highlighting how prolonged changes in network activity can translate into maladaptive circuit function (42). These observations support the broader view that chronic stress-induced pathology emerges not only from molecular adaptations but also from disruptions in circuit-level homeostasis. Acute increases in excitatory transmission are essential for adaptive learning and behavioral flexibility, but prolonged activation imposes substantial metabolic demands on neurons, requiring continuous adenosine triphosphate (ATP) production, calcium regulation, neurotransmitter recycling, and synaptic maintenance (7, 8, 44–46). When these demands persist over extended periods, compensatory mechanisms that normally restore network homeostasis become increasingly challenged (2, 5). As a result, chronic stress can initiate structural and molecular adaptations that reduce neuronal complexity and alter function.

A second mechanism involves disruption of activity-dependent neurotrophic signaling. Among the many candidates implicated in stress-related plasticity, BDNF occupies an important position given its role in supporting neuronal survival, dendritic growth, synapse formation, and long-term potentiation, which are processes necessary for healthy neural circuitry maintenance (10, 47). Indeed, several studies have consistently shown that chronic stress reduces BDNF expression within stress-sensitive brain regions (e.g., mPFC, HIPP; (48, 49), while reductions in BDNF signaling are associated with impaired learning, memory deficits and increased vulnerability to mood disorders (10, 50). For example, individuals carrying the *BDNF* val66met polymorphism exhibited impaired activity-dependent BDNF secretion, altered hippocampal function, and poorer episodic memory performance, highlighting the importance of intact neurotrophic signaling for adaptive plasticity (51). The coordinated nature of these adaptations raises a fundamental question, what neural mechanisms orchestrate these widespread changes across anatomically distinct brain regions?

Under this theoretical framework, chronic hyperactivation of OX pathways may contribute to the transition from adaptive plasticity to maladaptive remodeling, thereby increasing vulnerability to psychopathology (see **Figure 1**). As previously described, chronic stress repeatedly recruits CRF-sensitive OX neurons, while local glutamatergic feedback circuits amplify and sustain OX network activity (18, 32). Given that OX projections reach multiple corticolimbic structures simultaneously, prolonged stimulation of the OX system has the capacity to expose numerous brain regions to sustained excitatory and neuromodulatory influences such that this prolonged activation may increase metabolic burden, disrupt homeostatic regulation, and contribute to reductions in activity-dependent neurotrophic processes. Recent conceptual models support this interpretation as it has been proposed that OX participates in stress-related plasticity by altering activity-dependent transcriptional pathways regulating synaptic plasticity, cellular metabolism, stress adaptation, and neurotrophic signaling following repeated stress exposure (14). Rather than functioning exclusively as an arousal system, OX may therefore influence the way stress-responsive circuits adapt to environmental challenges over time.

Within the framework proposed here, persistent OX-dependent hyperarousal may represent one mechanism through which chronic stress maintains abnormal levels of neural activity across corticolimbic circuits, thereby increasing susceptibility to maladaptive neuroplastic remodeling. It should be noted, however, that this hypothesis does not imply OX functions independently of other biological systems. Certainly, glucocorticoids, inflammatory signaling pathways, monoaminergic neurotransmission, and glutamatergic mechanisms all are well-established contributors to stress-induced neuroplastic alterations (3, 5). Instead, OX may function as a convergent regulator that amplifies and coordinates stress-induced neuroadaptations across distributed neural networks. Such role would help explain how chronic stress produces remarkably similar patterns of structural and functional adaptations across multiple corticolimbic regions despite their distinct cellular and molecular characteristics.

An additional consideration is that OX neurons do not exclusively signal through OX peptides. Substantial evidence indicates that many OX neurons express vesicular glutamate transporter-2 and are capable of co-releasing glutamate at downstream targets (52, 53). Consequently, some effects attributed to OX transmission may reflect a combination of neuropeptide transmission and glutamatergic excitation. This distinction may be particularly relevant in the context of chronic stress because glutamate transmission plays a central role in activity-dependent plasticity, dendritic remodeling, and stress-induced adaptations within corticolimbic circuits. Thus, chronic recruitment of OX neurons may influence neural plasticity not only through OXR signaling but also through sustained glutamatergic drive, an important possibility that warrants further investigation.

Importantly, direct evidence that chronic OXR signaling independently drives the structural neuroplastic adaptation observed in the mPFC, HIPP, or AMY remains limited. While OX transmission has been shown to influence neuronal excitability, stress responsivity, fear expression, and motivated behavior, most evidence connecting OX to stress-induced dendritic remodeling, reductions in BDNF signaling, or impaired neurogenesis remains indirect (12, 26, 29). Consequently, the framework proposed here should be viewed as a testable mechanistic hypothesis in which OX functions as a potential amplifier or coordinator of neuroplastic adaptation initiated by multiple stress-responsive systems rather than a definitively established causal driver. Future studies directly examining whether chronic manipulations of OXR signaling alters structural plasticity will be necessary to clarify the role of OX in these processes.

## 5 Circuit-level consequences of chronic OX hyperactivation

Although chronic stress induces widespread neuroplastic adaptations throughout the brain, these changes are not uniformly distributed across neural systems (54). Instead, stress related remodeling tends to occur within a network of interconnected corticolimbic structures that collectively regulate emotional behavior, cognitive flexibility, memory processing, threat detection, and reward responsiveness (3, 5). Because OX neurons project broadly throughout these regions and influence neuronal excitability across multiple neurotransmitter systems, persistent OX-dependent hyperarousal has the potential to coordinate stress-induced adaptations across distributed neural circuits rather than affecting individual structures in isolation (12, 17, 20).

### 5.1 Medial prefrontal cortex: loss of top-down regulation

The mPFC plays a critical role in executive function, emotional regulation, decision making, and inhibitory control of stress-responsive neural systems (55). Under normal conditions, the mPFC exerts top-down regulation over subcortical circuits involved in fear, arousal, and autonomic activation, thereby contributing to adaptive responses during stressful situations (3, 5). Chronic stress, however, produces profound structural and functional alterations within this region that impair its regulatory capacity. Using intracellular dye-filling techniques in adult male Sprague-Dawley rats, 21 days of repeated restraint stress produced approximately 20% reductions in apical dendritic length and 17% reductions in dendritic branching in layer II/III pyramidal neurons of the mPFC (7). These structural alterations were selective for apical dendrites, suggesting that chronic stress preferentially affects inputs involved in cortical integration and higher-order information processing. It was further proposed that such dendritic remodeling may impair prefrontal inhibitory control of the hypothalamic-pituitary-adrenal HPA axis, thereby promoting prolonged physiological responses to stress (7).

These findings are particularly relevant within the context of OX-dependent hyperarousal. OX neurons project directly to the mPFC, where both OX1R and OX2R are expressed on cortical neurons (12, 28). Both receptor subtypes appear to contribute to cortical excitability, although available evidence suggests that OX1R signaling may play a particularly important role in regulating pyramidal neuron activity and cortical glutamatergic transmission (12, 56). OX receptors primarily signal through Gq/11-dependent pathways that increase intracellular calcium and promote neuronal depolarization, thereby enhancing cortical responsiveness to incoming stimuli (12, 17). It was recently demonstrated in mice that activation of the lateral hypothalamic OX-mPFC pathway contributed to chronic stress-induced anhedonia (28), and chemogenetic inhibition alleviated it, indicating that excessive OXR signaling can influence prefrontal function (56, 57). These outcomes suggest that chronic recruitment of OX transmission may contribute to stress-induced prefrontal dysfunction by maintaining prolonged excitatory drive within a region already vulnerable to structural remodeling. However, direct evidence demonstrating that chronic OXR signaling independently produces the dendritic alterations observed following chronic stress remains limited. Thus, OX may function as an amplifier of prefrontal vulnerability rather than a sole driver of structural remodeling. Interestingly, diminished prefrontal regulation may represent an important neuroadaptation through which chronic stress compromises emotional regulation and increases vulnerability to affective dysfunction (5). As structural integrity declines, the capacity of the mPFC to suppress stress-responsive subcortical systems becomes compromised, potentially creating a feed-forward cycle in which heightened OX activity further weakens cortical regulation while simultaneously promoting ongoing arousal. Reduced prefrontal inhibitory control may also contribute to persistent activation of stress-responsive CRF circuits, thereby facilitating continued recruitment of OX neurons and reinforcing an allostatic cycle of hyperarousal (5, 7, 46). Such reciprocal interactions may help explain how transient stress responses evolve into persistent neurobiological states associated with depression- and anxiety-related psychopathology.

### 5.2 Hippocampus: impaired neuroplasticity and contextual regulation

The HIPP plays a central role in learning, memory, contextual processing, and negative feedback regulation of the HPA axis (58, 59). The HIPP is not functionally homogeneous. The dorsal HIPP is primarily involved in spatial learning, memory, and cognitive processing, whereas the ventral HIPP maintains extensive connectivity with the mPFC, AMY, and hypothalamus and plays a more prominent role in emotional regulation and anxiety-related behavior (60, 61). Given these connections, OX-dependent modulation of ventral HIPP circuitry may be particularly relevant to the affective consequences of chronic stress. However, whether chronic OX activation differentially influences dorsal versus ventral HIPP plasticity remains largely unknown. Among corticolimbic structures, it is one of the most consistently affected by chronic stress. Across multiple rodent models, long-lasting exposure to stress and glucocorticoid administration produce dendritic atrophy, synaptic reorganization, and reduces neurogenesis within hippocampal circuitry (9, 62–64). Collectively, these adaptations impair hippocampal function and reduce the ability of the organism to appropriately contextualize and regulate stress responses. Several observations suggest that OXR signaling may contribute to these vulnerabilities. OXRs are expressed throughout the HIPP, where OX peptides influence neuronal excitability, synaptic transmission, and memory-related processes (65). Although under normal acute stress conditions, OXR signaling may facilitate learning and attentional processes, sustained activation of OXRs could impose lasting excitatory demands that interact with glucocorticoid-mediated suppression of neuroplasticity (66–68). Consistent with this possibility, chronic stress reduces hippocampal BDNF expression (50, 66), while impaired activity-dependent BDNF signaling is associated with deficits in hippocampal function and episodic memory (51). Taken together, these findings suggest that persistent CRF→OX recruitment may contribute to an environment in which adaptive hippocampal plasticity becomes increasingly difficult to maintain. Nevertheless, direct evidence demonstrating that chronic OXR signaling causes hippocampal dendritic atrophy, impaired neurogenesis, or reductions in BDNF expression remains limited. Accordingly, OX may be better conceptualized as a modulatory factor that interacts with established glucocorticoid- and stress-related plasticity mechanisms.

### 5.3 Amygdala: enhanced threat detection and emotional reactivity

In contrast to the mPFC and HIPP, chronic stress typically increases structural complexity in the AMY, which plays a central role in fear conditioning, threat detection, and emotional salience, making it particularly relevant to stress-related psychopathology (69). In adult male Wistar rats exposed to chronic immobilization stress, significant dendritic hypertrophy within the basolateral AMY was observed (8). Unlike the stress-induced atrophy observed in the HIPP, chronic stress increased dendritic arborization and branching in the AMY, suggesting enhanced capacity for emotional and threat related information. Critically, these structural changes were accompanied by increased anxiety-like behavior in the elevated plus maze, linking amygdalar remodeling to behavioral outcomes (8).

OX appears well-positioned to influence these adaptations, as OX fibers innervate both the central AMY and bed nucleus of the stria terminalis, regions heavily involved in fear and anxiety processing (12). Indeed, OX1Rs are abundantly expressed within amygdalar circuits and appear to mediate many of the anxiogenic and fear-related effects of OX transmission. Electrophysiological studies have demonstrated OX-A depolarizes AMY neurons through OX1R-dependent mechanisms involving phospholipase-C activation, intracellular calcium mobilization, and sodium-calcium exchanger activity, ultimately promoting neuronal excitability and conditioned fear expression (26). Furthermore, pharmacological OX1R blockade attenuated these effects, indicating that OX1R signaling contributes directly to threat-related neural processing. Thus, persistent activation of OX-dependent inputs may reinforce stress-induced amygdalar plasticity by maintaining elevated neuronal activity within fear-related circuits. However, whether chronic and persistent activation of OXR signaling directly drives the structural hypertrophy observed following prolonged stress exposure remains unknown and will require direct experimental investigation.

### 5.4 Mesolimbic reward circuits: anhedonia and motivational dysfunction

Beyond fear and stress regulation, chronic stress frequently disrupts reward processing and motivational behavior, which are particularly relevant to depression, where diminished reward sensitivity and anhedonia represent core clinical features (70). Still, compared with stress-induced remodeling of the mPFC, HIPP, and AMY, direct evidence linking chronic OX activation to structural neuroplastic changes within mesolimbic circuitry remains comparatively limited. Available evidence suggests OX neurons project directly to the VTA, a key component of the mesolimbic reward system. Within the VTA, OX transmission appears to influence both dopaminergic and GABAergic neuronal populations, providing multiple mechanisms through which OX may regulate reward processing (29, 71). While the relative contributions of OX1R and OX2R signaling within the mesolimbic circuitry remain characterized incompletely, evidence suggests that OX1R-mediated modulation of dopaminergic transmission may be particularly relevant to motivated behavior and reward seeking (12, 29). Although acute activation of this pathway may facilitate adaptive motivational behavior, chronic recruitment of the system may produce different outcomes. Sustained elevations in dopaminergic signaling can trigger compensatory adaptations that reduce reward sensitivity over time, including receptor desensitization and altered downstream signaling pathways (72, 73). Chronic OX hyperactivation may influence depression not only through stress and anxiety pathways, but also through disruption of reward circuitry. Consequently, OX-dependent hyperarousal has the potential to induce multiple issues simultaneously, including emotional dysregulation, anxiety, cognitive dysfunction, and anhedonia.

### 5.5 Integrating circuit-level adaptations

Although chronic stress produces region specific adaptations, a common pattern emerges across corticolimbic circuitry. Neural systems responsible for cognitive control and contextual regulation, including the mPFC and HIPP, exhibit reductions in structural complexity and neuroplastic capacity (74). In contrast, circuits involved in threat detection and emotional salience, such as the AMY, become increasingly reactive (8). Simultaneously, alterations within mesolimbic reward pathways contribute to diminished reward sensitivity and motivational dysfunction (72, 73). Thus, we propose that persistent OX-dependent hyperarousal may help coordinate these distributed adaptations. By maintaining elevated activity across stress-sensitive neural networks, chronic OX recruitment may contribute to the transition from adaptive stress responsiveness to enduring circuit-level neuroadaptations that increase vulnerability to depression- and anxiety-related disorders. The potential result may be a brain increasingly biased toward vigilance, threat monitoring, and negative affect while becoming progressively less capable of flexible cognitive and emotional regulation, a neurobiological profile that closely mirrors the symptomatology of depression and anxiety disorders.

Although the framework proposed here positions OX as a potential coordinator of these distributed adaptations, it is important to note that direct evidence linking chronic OX activation to the structural remodeling observed across corticolimbic regions remains incomplete. Much of the existing literature demonstrates effects of OX transmission on arousal, neuronal excitability, emotional processing, and motivated behavior, whereas its role in long-term morphological plasticity remains largely inferential. Therefore, OX should presently be viewed as a candidate amplifier of stress-induced neuroadaptations rather than a definitively established causal factor.

## 6 Developmental and individual vulnerability factors

The neuroplastic consequences of chronic stress are not uniformly expressed across individuals. Exposure to comparable stressors can produce substantially different behavioral and neurobiological outcomes depending on developmental stage, biological sex, genetic background, and preexisting circuit organization (75). These factors influence the degree to which stress-responsive systems are recruited and may ultimately determine whether stress-induced neuroplastic adaptations remain adaptive or progress toward psychopathology. Furthermore, they may also influence the extent to which CRF-sensitive OX pathways contribute to the development of prolonged arousal states and maladaptive circuit remodeling.

### 6.1 Developmental vulnerability

Adolescence represents a particularly sensitive period for stress-induced neuroplastic disruption because corticolimbic circuits continue to undergo substantial maturation throughout this developmental window (76–78). In juvenile male Sprague Dawley rats exposed to 4 weeks of chronic variable stress, persistent reductions in hippocampal growth, altered glucocorticoid receptor expression, deficits in spatial learning, and impaired regulation of stress-induced corticosterone secretion that remained evident into adulthood were observed (79). These findings suggest that stress exposure during adolescence can alter neural maturation rather than simply produce transient physiological disruption. Because the OX system contributes to arousal and emotional regulation, and HPA-axis function, developmental disturbances in OX signaling may have long-lasting consequences for stress vulnerability. Consistent with this possibility, early-life manipulations of OX signaling have been shown to influence emotional behavior and stress responsivity later in life, suggesting that developmental recruitment of OX transmission may have long-term consequences for psychiatric vulnerability (80, 81). Thus, adolescence may represent a period during which repeated CRF-dependent activation of OX neurons exerts particularly enduring effects on neural plasticity and behavioral outcomes.

### 6.2 Biological sex

Biological sex is another important determinant of vulnerability to stress-related psychopathology (22). In the past decades, studies have consistently demonstrated that anxiety disorders, major depressive disorder, and trauma-related disorders occur more frequently in women than in men (82). Using adult male and female Sprague-Dawley rats, it was demonstrated that CRF receptors exhibited greater coupling to stimulatory G-proteins in females and remained more responsive following repeated stress exposure because receptor internalization occurred predominantly in males (83). Because CRF directly recruits OX neurons within the LH (18), prolonged CRF sensitivity may amplify OX activation during chronic stress and increased susceptibility to downstream neuroplastic adaptations. Although sex-specific OX mechanisms remain to be completely understood, available evidence suggests that interactions between CRF and OX systems may contribute to sex differences in stress-related psychopathology.

### 6.3 Genetic vulnerability

Genetic variation also influences susceptibility to stress-induced neuroplastic dysfunction. One of the most extensively studied examples is the BDNF *Val66Met* polymorphism. Individuals carrying the Met allele exhibit impaired activity-dependent BDNF secretion, reduced hippocampal activation, and poorer episodic memory performance relative to Val homozygotes (51). Within the context of chronic stress, such impairments may reduce the capacity of neural circuits to maintain adaptive plasticity and increased vulnerability to stress-related pathology. Genetic influences may also operate through inhibitory regulation of OX neurons. Variants associated with reduced GABAergic signaling have been linked to panic-related phenotypes (13), and diminished inhibitory tone could lower the threshold for OX recruitment during chronic stress (84). Consequently, genetic background may influence both the magnitude of OX activation and the long-term neuroplastic consequences of sustained arousal.

### 6.4 Resilience and individual differences

Not all individuals exposed to chronic stress develop depression or anxiety disorders, indicating that resilience mechanisms actively oppose maladaptive neuroplastic remodeling (85–87). Although resilience is multifactorial, evidence indicates that maintenance of prefrontal regulatory function, preservation of BDNF signaling, intact inhibitory control, and efficient recovery of stress-response systems promote adaptive outcomes following adversity (88, 89). From this perspective, resilience may reflect the ability to limit the accumulation of stress-induced neuroadaptations by efficiently attenuating CRF- and OX-mediated arousal signaling once environmental threats have subsided. Individuals capable of restoring homeostatic balance may avoid the prolonged recruitment of CRF-responsive OX neurons necessary to initiate widespread circuit remodeling, whereas persistent activation of CRF systems may sustain OX transmission throughout stress-sensitive corticolimbic networks. Such prolonged recruitment may promote chronic vigilance, emotional dysregulation, and progressive neuroplastic adaptations that increase vulnerability to depression- and anxiety-related psychopathology. Conversely, resilience may be associated with more efficient regulation of CRF and OX activity, thereby reducing the duration and magnitude of stress-induced arousal and limiting downstream neuroplastic dysfunction.

## 7 Therapeutic implications: targeting hyperarousal and restoring neuroplasticity

The model proposed here suggests that chronic stress-induced psychopathology emerges not simply from exposure to adversity but from the persistent recruitment of neural systems that maintain hyperarousal and promote neuroplastic dysfunction. Under this model, effective interventions should ideally achieve one or both of two therapeutic goals: (1) reducing pathological activation of stress-responsive arousal networks and (2) restoring adaptive plasticity within corticolimbic circuits. These objectives are not mutually exclusive, and several emerging therapeutic approaches may influence both simultaneously.

### 7.1 Direct modulation of the OXR signaling

The OX system has emerged as an attractive therapeutic target because of its central role in arousal, vigilance, autonomic activation, and emotional responsivity. OXR antagonists were initially developed for the treatment of insomnia, but growing evidence suggests that these compounds may also influence affective processes relevant to depression and anxiety (90, 91). Dual orexin receptor antagonists (DORAs), which block both OX1 and OX2 receptors, reduce hyperarousal by suppressing OX-mediated activation of wake-promoting and stress-responsive neural systems (92). In this context, clinical studies examining the efficacy of the DORA daridorexant have demonstrated reductions in neurophysiological markers of hyperarousal and improvements in sleep continuity among individuals with chronic insomnia (93). Given the strong relationship between chronic stress, sleep disruption, and psychiatric vulnerability, attenuation of pathological arousal may represent an important mechanism through which DORAs exert broader therapeutic effects.

Single orexin receptor antagonists (SORAs) provide an additional strategy for targeting OX-related dysfunction. Because OX1 and OX2 receptors differ in their anatomical distribution and behavioral functions, selective OXR blockade has the potential to modulate specific aspects of stress-related pathology while minimizing unwanted side effects (94, 95). For instance, OX1R signaling appears particularly relevant to anxiety, fear expression, and stress reactivity, whereas OX2R signaling contributes more strongly to arousal and sleep-wake regulation (12). This distinction raises the possibility that receptor-interventions could be tailored to specific symptom dimensions (OX1R vs. OX2R). Importantly, recent clinical investigations have begun evaluating OX receptor antagonists as either adjunctive or monotherapy treatments for depression, particularly in patients exhibiting insomnia and hyperarousal symptoms (96, 97). Although additional work is required to determine their long-term efficacy, these findings support the view that interventions targeting OXR signaling may influence psychiatric outcomes beyond sleep regulation.

### 7.2 Sleep as a therapeutic target

A recurring theme throughout this review is the close relationship between hyperarousal, OX signaling, and sleep disruption. Sleep disturbances are among the most common symptoms observed in stress-related psychiatric disorders and frequently persist even after improvements in mood symptoms (98). Because OX neurons play a central role in regulating wakefulness, chronic activation of OX transmission may contribute directly to impaired sleep quality and prolonged physiological arousal (99). Interventions that restore healthy sleep patterns may therefore interrupt the self-reinforcing cycle linking stress, hyperarousal, and neuroplastic dysfunction. In this context, OX receptor antagonists, behavioral sleep interventions, and emerging neuromodulation approaches may provide complementary strategies for reducing arousal burden and facilitating recovery of neural function. Conversely, the recent development of OXR agonists for disorders characterized by deficient OX transmission (see for example, 100, 101) further underscores the central role of the OX system in regulating arousal and sleep-wake stability; however, the therapeutic implications of these compounds for stress-related psychopathology remain largely unexplored.

### 7.3 Alternative mechanisms and future directions

Although chronic CRF-dependent activation of OX neurons is proposed to contribute to prolonged arousal and maladaptive neuroplastic remodeling, stress-related psychopathology is unlikely to arise from any single biological mechanism. Chronic stress engages multiple interacting systems, including glucocorticoid signaling, neuroinflammatory pathways, glutamatergic transmission, and monoaminergic networks, each of which has been independently implicated in depression- and anxiety-related disorders (2–5, 42). Emerging evidence also suggests that alterations in excitation-inhibition balance within stress-responsive circuits may represent an important mechanism contributing to stress-related psychopathology. For example, chronic stress-induced depression-like states have been linked to persistent excitation-inhibition imbalance within MPOA circuitry, demonstrating how circuit-level dysregulation can contribute to the emergence of affective disturbances (42). In this context, OX transmission may influence psychopathology not only through direct receptor-mediated effects but also by interacting with broader mechanisms regulating circuit excitability and network stability. Accordingly, OX should not be viewed as an isolated causal factor but rather as a stress-responsive network that may amplify and coordinate the influence of these systems across corticolimbic circuitry. Furthermore, much of the evidence reviewed here derives from rodent studies, frequently conducted in male animals, limiting the extent to which current findings can be generalized across sexes, strains, and human populations.

Despite these limitations, converging anatomical, molecular, and behavioral evidence supports continued investigation of OX signaling as a contributor to stress-induced neuroplastic dysfunction. A major unresolved question is whether chronic OX activation functions primarily as a causal driver of circuit remodeling, an amplifier of alterations initiated by other stress-responsive systems, or a biomarker of ongoing pathological hyperarousal. At present, the literature more strongly supports a role for OX transmission in regulating arousal, stress responsivity, and circuit excitability than in directly producing structural neuroplastic adaptations. Consequently, one of the most important unresolved questions is whether OX contributes causally to stress-induced dendritic remodeling and neurotrophic dysfunction or instead functions as a permissive or amplifying mechanism through which alterations initiated by glucocorticoid, inflammatory, and glutamatergic systems are propagated across distributed neural networks. Distinguishing among these possibilities represents a critical next step for evaluating the validity of the framework proposed here. Future studies combining circuit-specific manipulation of orexin pathways with longitudinal assessments of structural and molecular plasticity will be critical for addressing this question. Particular attention should be devoted to sex differences, developmental vulnerability, and translational biomarkers capable of linking preclinical findings with human stress-related psychopathology. Importantly, the framework proposed here generates several experimentally testable predictions. If persistent OX-dependent hyperarousal contributes directly to stress-induced neuroplastic dysfunction, then pharmacological or circuit-specific inhibition of OX signaling during chronic stress exposure should attenuate reductions in hippocampal BDNF expression, preserve mPFC dendritic architecture, limit stress-induced amygdalar hypertrophy, and ultimately reduce the emergence of anxiety-like and depression-like behavioral phenotypes. Conversely, prolonged activation of OX pathways in the absence of chronic stress should be sufficient to reproduce at least some components of this neuroplastic profile. Clarifying these relationships may ultimately determine whether interventions targeting OX signaling can modify the trajectory of depression- and anxiety-related disorders before persistent circuit dysfunction becomes established.

## 8 Conclusion

Chronic stress produces widespread neuroplastic adaptations throughout corticolimbic circuitry that contribute to the development of depression- and anxiety-related disorders. While these adaptations have traditionally been examined through the lenses of glucocorticoid signaling, neurotrophic dysfunction, and neurotransmitter imbalance, growing evidence suggests that OX signaling may represent an important intermediary between stress exposure and long-term circuit remodeling. Because OX neurons receive direct input from stress-responsive CRF systems and project broadly to brain regions involved in cognition, emotion, fear, and reward, persistent orexin-dependent hyperarousal may help coordinate the distributed neural adaptations observed following chronic stress. We propose that chronic CRF→OX recruitment contributes to neuroplastic adaptations within the mPFC, HIPP, AMY, and mesolimbic reward system that may collectively increase vulnerability to depression- and anxiety-related disorders (see **Figure 1**). Although OX is unlikely to represent the sole mechanism underlying stress-related psychopathology, the evidence reviewed here is consistent with a model in which OX signaling functions as an allostatic amplifier that extends the influence of chronic stress across multiple neural systems, promoting enduring neuroadaptations that may ultimately increase vulnerability to depression- and anxiety-related psychopathology.
